# Newborn hearing screening program in China: a narrative review of the issues in screening and management

**DOI:** 10.3389/fped.2023.1222324

**Published:** 2023-09-05

**Authors:** Cheng Wen, Li-Hui Huang

**Affiliations:** ^1^ Department of Otolaryngology—Head and Neck Surgery, Beijing Tongren Hospital, Capital Medical University, Beijing, China; ^2^ Beijing Institute of Otolaryngology, Beijing, China; ^3^ Key Laboratory of Otolaryngology Head and Neck Surgery, Ministry of Education, Beijing, China

**Keywords:** newborn hearing screening, hearing loss, genetic screening, children, concurrent screening

## Abstract

Hearing loss is one of the most common sensory disorders in humans. The purpose of this review is to summarize the history and current status of newborn hearing screening in China and to investigate future developmental trends in newborn hearing screening with the intention of sharing experiences and providing a reference for other populations. In the 1980s, the research on hearing monitoring for high-risk infants led to the gradual development of newborn hearing screening in China. With the continuous improvement of screening technology, the newborn hearing screening program was gradually extended to the whole country and became a government-led multidisciplinary public health program. Genetic screening for deafness has been incorporated into newborn hearing screening in many regions of China to help screen for potential and late-onset deafness in newborns. In the future, it is necessary to further establish and improve whole life-cycle hearing screening and healthcare, conduct screening for congenital cytomegalovirus infection, and create a full-coverage, whole life course hearing screening and intervention system. Screening for deafness in China has been marked by 40 years of achievements, which have been a source of pride for entrepreneurs and comfort for patients and their families. Managing hearing screening data information more efficiently and establishing a quality control index system throughout the whole screening process are of paramount importance. The genetic screening for concurrent newborn hearing and deafness has a great clinical importance for the management of congenital deafness and prevention of ototoxicity. A hearing screening and intervention system across the whole life course should be developed.

## 1. Introduction

Hearing loss is one of the most common congenital disabilities worldwide. The World Report on Hearing published by the World Health Organization (WHO) indicates that >1.5 billion people currently experience some degree of hearing loss, a number that could grow to 2.5 billion by 2050 (1). The WHO estimates that more than 400 million people, including 34 million children, live with disabling hearing loss, which affects their health and quality of life (1). The global prevalence of moderate-to-severe hearing loss increases with age from 0.2% in early neonates to 1.5% in children aged 5–9 years (1). The impact of hearing loss on children is dependent on age at onset and severity; thus, early detection is a necessity. Delaying hearing tests negatively affects growing children in terms of delayed language acquisition, speech development, literacy, and social skills (2).

Universal newborn hearing screening (UNHS) has been widely and intensively implemented in China, enabling early detection, diagnosis, and intervention for children with hearing loss (3). Before the implementation of this newborn hearing screening, a localized research reported that the average age of hearing loss detection in children was approximately 24–28 months (4). The average age of detection in rural areas was approximately 8.63 months later than that in urban areas. Even in children with bilateral hearing loss in rural areas, only 54.10% received hearing intervention, and 69.70% received no speech training after the hearing intervention (5). After the hearing screening program was conducted, the average age at which hearing loss was confirmed dropped to 3–4 months (6). A cost-effectiveness analysis showed that a universal hearing screening strategy using otoacoustic emissions (OAEs) averted 7,310 disability-adjusted life years (DALYs) and a universal hearing strategy using OAEs plus automatic auditory brainstem response (AABR) averted 7,710 DALYs (7). Hearing screening programs and follow-up with hearing aids or other treatments as appropriate were reported to be cost-effective from 2005 to 2009 in five of eight provinces in China, China as a whole (8). With early detection (within 1 month), diagnosis (within 3 months), and interventions (within 6 months), together with scientific auditory speech rehabilitation, children with hearing loss could achieve normal or near-normal speech development and eventually integrate into mainstream society.

In the 1980s and 1990s, audiological techniques such as auditory brainstem response (ABR) tests, transient evoked otoacoustic emissions (TEOAEs), and distortion product otoacoustic emissions (DPOAEs) led to the gradual development of newborn hearing screening in China. In 2004, the former Ministry of Health issued the Technical Specification for Newborn Hearing Screening, which set clear requirements for screening institutions, personnel, housing, and equipment, as well as for hearing screening, diagnosis, intervention, and quality control (9). With the gradual expansion of newborn hearing screening across the country, much concern about the standardization of screening technology and quality was investigated. In 2009, the Draft Guidelines for Early Hearing Detection and Intervention in Newborns and Infants was published in the *Chinese Journal of Otolaryngology Head and Neck Surgery* (10), thus promoting the revision and introduction of the Technical Specification for Newborn Hearing Screening (2010 Edition) [hereinafter referred to as the “Specification (2010 Edition)”] by the former Ministry of Health (11). Since its implementation, the Specification (2010 Edition) has played a beneficial role in the standardization of screening technology and the improvement of screening quality. Wen et al. (2) conducted a quality assessment and analysis of universal newborn hearing screening guidelines and found that the Specification (2010 Edition) achieved an overall score of 5 in China, and recommendations were consistent with international mainstream opinions. The public health project is led by the government with collaborators in the perinatal, child health, audiology, otolaryngology, applied electronics, pediatrics, psychology, social, and educational disciplines, and this project has played a pioneering role in promoting the development of audiology in China.

Causative factors that lead to hearing loss during the prenatal period include genetic factors, intrauterine infections, hypoxia or birth asphyxia, low birth weight, hyperbilirubinemia, and other perinatal morbidities (1). Genetic factors are responsible for more than 50% of hearing loss encountered in neonates and account for nearly 40% of hearing loss during childhood (1). With the understanding of the etiology of neonatal hearing loss, genetic factors have received increasing attention. Beijing took the lead in launching a large-scale genetic screening program for neonatal deafness in 2012, which in turn promoted the development of concurrent newborn hearing and genetic screening programs. In addition, clinical and basic medical research related to delayed hearing loss in children in China promoted the promulgation of the Technical Specification for Children's Ear and Hearing Care by the former Ministry of Health in 2013 (12).

**Figure 1:**
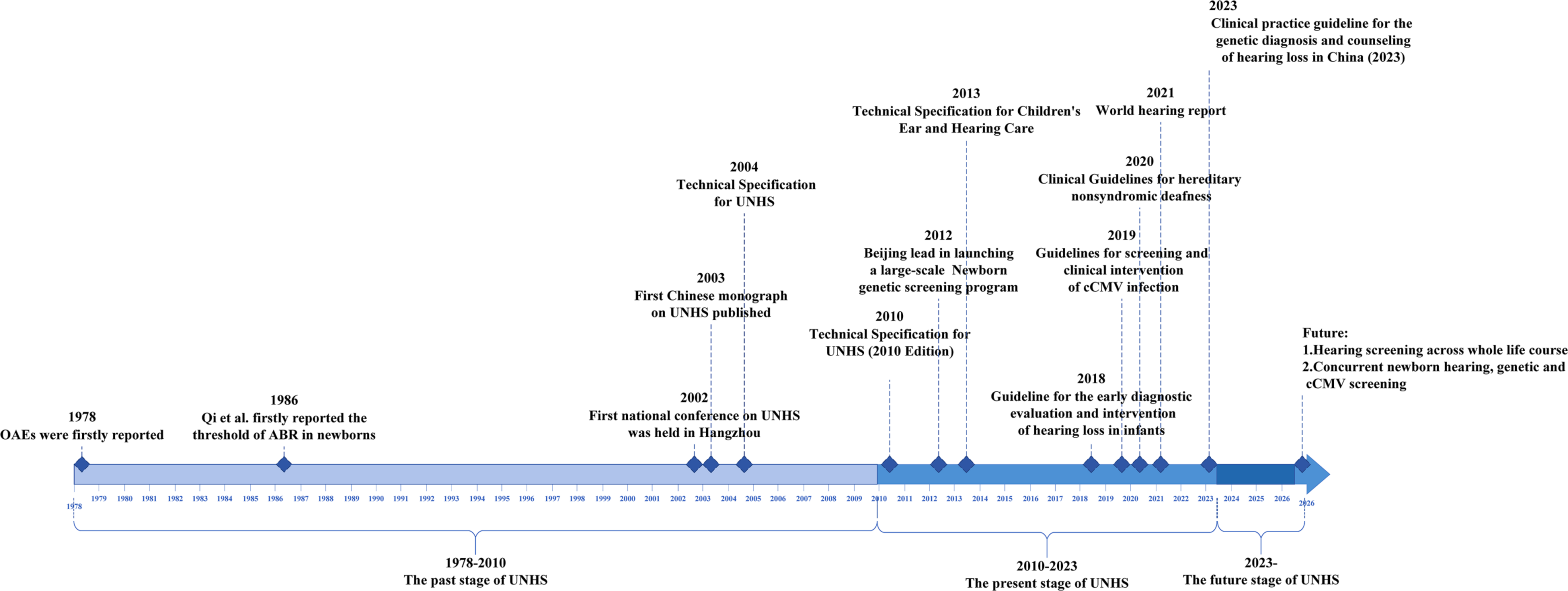
Timeline chart of newborn hearing screening.

Looking back over the previous four decades, we have a deep understanding of the difficulties encountered in the first stages of the development of newborn hearing screening program in China, and the results were achieved therein. However, many of the early important research results on newborn hearing screening in China had only been published in the Chinese literature. We write this review to help domestic and foreign scholars understand the changes in newborn hearing screening programs in China. Its purpose is to summarize the history and current status of the newborn hearing screening in the country and to investigate future developmental trends with the intention of sharing experiences and providing a reference for other regions. As presented in Figure 1, the history of newborn hearing screening in China over the past 40 years can be summarized into three stages: 1978–2010, the past stage of the newborn hearing screening; 2010–2023, the present stage of the newborn hearing screening; and 2023 onward, the future stage of the newborn hearing screening.

## 2. The past stage of newborn hearing screening

In the early 1980s, when many newborns faced with the dilemma of hearing loss, newborn hearing screening in China gradually expanded from localized to nationwide coverage, realizing the transition from hearing monitoring for high-risk infants to universal newborn hearing screening. The continuous update of screening technology has formed the current model in China.

### 2.1. Early research on newborn hearing screening

Yisheng Qi was the first to carry out exploratory newborn hearing screening in China in the early 1980s. In 1986, Qi et al*.* (13) reported the ABR threshold in newborns for the first time and noted that the ABR characteristics in newborns could be a reliable indicator for the clinical diagnosis of hearing impairment. In 1987, Qi et al. (14) were the first to carry out hearing monitoring for high-risk newborns in China, which provided new indicators for clinical ABR tests and for predicting the development of hearing and neurological function in high-risk neonates. In 1988, studies showed that it was feasible to perform acoustic conductance testing on neonates, and the literature on the pediatric ABR was summarized for clinical application (15, 16). OAEs were first reported by Kemp et al. (17) in 1978 and were gradually applied to newborn hearing screening in other countries in the 1990s (18, 19). In 1994, Wang et al*.* (20) found that TEOAEs were faster and more convenient than ABR tests and could be a useful method to screen newborns for hearing impairment. Nie et al. showed that TEOAEs could be used as a monitoring method for the early detection of pediatric hearing loss and later reported that no significant sex difference in response energy across the TEOAE frequency bands in full-term neonates was found (21, 22).

In the 1980s and 1990s, Chinese scholars used ABR tests and TEOAEs to conduct exploratory studies of newborn hearing detection, clarified the response characteristics of ABR tests and TEOAEs in newborns, and concluded that while both tests have an application value in neonatal hearing detection, TEOAEs are more adequate for rapid screening.

### 2.2. Universal newborn hearing screening

In 1999, Liao et al*.* (23) used TEOAEs and DPOAEs to screen 108 newborns and compared their ABR test results. They found that OAEs were a rapid and effective method for newborn hearing screening. In the same year, Zhang et al*.* (24) performed TEOAE screening on 2,796 newborns and found that an initial screening at 2–3 days after birth was more appropriate. In 1999, the study by Nie et al*.* (25) was the first to report the overall incidence of congenital hearing loss in Beijing, in which the incidence of hearing loss was more than 2.04% in both directions, making the first study of regional newborn hearing screening in China. In 2003, Ni and Gu (26) proposed the population, purpose, and methods of newborn hearing screening, and Bu (27) emphasized that newborn hearing screening is a systematic project that includes screening, diagnosis, intervention, follow-up, and quality assessment and considered that accurate diagnosis is particularly important. Qi and Huang (28) pointed out the development direction of newborn hearing screening in China and provided detailed suggestions on how to implement newborn hearing screening. In September 2002, the first national conference on neonatal hearing screening was held in Hangzhou, Zhejiang Province, jointly sponsored by the Editorial Board of the *Chinese Medical Journal* and the Zhejiang Health Department. A total of 160 colleagues from 21 cities attended the conference and reached a consensus on issues related to neonatal hearing screening and diagnosis. This conference promoted a well-organized development of hearing screening and became one of the landmark events of newborn hearing screening in China (29). In 2003, the first domestic monograph on newborn hearing screening, Newborn and Infant Hearing Screening, which was edited by Han Demin, was published by the People's Medical Publishing House. In the same year, Huang et al. (30) held the first training course on newborn hearing screening in Beijing. In 2004, the national training textbook “Newborn Hearing Screening,” which was edited by Shen Xiaoming, was published by the People's Medical Publishing House. In the same year, Bu (31) called for the development of the “Principles and Guidelines for Early Hearing Detection and Intervention Programs” and for the definition revision of hearing disability and the classification of hearing loss in China.

To date, a system for newborn hearing screening and diagnosis has gradually been formed and more commonly promoted in major cities across the country.

### 2.3. Universal newborn hearing screening in rural and grassroots areas

In 2004, Cai et al. (32) pioneered a study on the feasibility of newborn hearing screening in rural areas. In the same year, Zhang (33) pointed out that the screening model of appointments with pediatricians at the township level and the establishment of review and referral lines from one level to another was applicable at the rural primary level. In 2005, Xiao et al. (34) found that the application of OAEs for newborn hearing screening in primary hospitals could detect hearing loss early. In 2006, Cai et al. (35) noted that newborn hearing screening in rural areas was feasible and necessary and called for the immediate establishment and improvement of newborn hearing screening models in rural areas. In the same year, Yang et al. (36) found that infants with different degrees of hearing loss accounted for 4.69% of the total number of screened infants, indicating the necessity of conducting newborn hearing screening at the primary level. In 2007, Wang and Bing (37) conducted a study of 2,535 newborns who underwent hearing screening and found that the primary screening pass rate was 88.99%, indicating that it was feasible to conduct newborn hearing screening at the primary level. In 2010, Qi et al. (38) investigated and analyzed newborn hearing screening in the combined urban‒rural area of Beijing, where a large floating (non-resident) population exists, and found that the rescreening and referral rates of UNHS were lower in the rural area than in the urban area. Therefore, developing a hearing screening model that is suitable for the characteristics of the floating population is important and necessary.

The above study suggests that newborn hearing screening is feasible and necessary in rural and grassroots areas. With the impetus of a national policy, newborn hearing screening is also gradually being implemented in rural and primary areas through continuous medical education in various regions.

## 3. The present stage of newborn hearing screening

At this stage, progress in three aspects, namely, standardized application of newborn hearing screening, concurrent newborn hearing and genetic screening, and children's hearing healthcare, indicates that the level of newborn hearing screening in China has entered the mainstream of international development.

### 3.1. Standardized application of newborn hearing screening

OAEs and AABR tests are the recommended hearing screening techniques for newborns worldwide (11, 39). In China, the “Specification (2010 Edition)” specified that OAEs or AABR tests should be used for newborn hearing screening and that AABR screening should be performed before discharge from the neonatal intensive care unit (NICU). Infants in the NICU who fail the initial screening test should be referred for further hearing diagnosis (11). In addition, several studies have shown that OAEs combined with AABR tests are effective in reducing the false-positive and false-negative rates in newborn hearing screening (40, 41). In 2019, Wen et al. (42) performed a meta-analysis of the sensitivity and specificity of OAEs and AABR tests, indicating that the combination of these tests was recommended in UNHS. The newly published Chinese clinical practice guidelines for auditory neuropathy (2022 edition) stated that infantile auditory neuropathy is often detected by combined OAEs and AABR screening, thus emphasizing that the correct choice of newborn hearing screening techniques is crucial for the early detection of auditory neuropathy (43). In 2012, Huang et al. (3) conducted a cost-effectiveness analysis of neonatal hearing screening programs in China, indicating that the universal screening is possibly considered the prioritized implementation goal, especially in relatively developed provinces, while it is possible that targeted screening is temporarily more realistic than the universal screening in developing provinces.

According to “Hearing screening and diagnostic management for children aged 0–6 years in Beijing,” healthcare institutions should refer children with suspected hearing loss detected by screening to six designated “Beijing Children's Hearing Diagnosis Medical Institutions” for confirmation and intervention. Community health service centers, district healthcare departments, and rural health centers within the administrative area should assist in following up children with suspected hearing loss. Rehabilitation institutions are responsible for providing family training and recovery for children with hearing loss (44). Hearing aid fitting is an important approach for early hearing intervention in infants (45). For infants with severe or profound sensorineural hearing loss (SNHL), cochlear implantation is generally recommended at approximately 12 months of age but may be performed earlier or later in some special circumstances (45). Infants with auricular abnormalities, atresia, or severe narrowing of the ear canal could wear soft band bone conduction hearing aids (45). Importantly, intervention effectiveness should always be assessed throughout the process of auditory speech rehabilitation.

### 3.2. Standardized management of newborn hearing screening

The government attaches importance to the prevention and treatment of hearing birth defects and has introduced a series of initiatives to promote the standardized implementation of newborn hearing screening. The National Health Commission of the People's Republic of China issued “Management of neonatal screening,” which explicitly stipulated that health administrative departments of provinces, autonomous regions, and municipalities directly under the central government were responsible for supervision and management of neonatal screening in the administrative region (46). In addition, each province, autonomous region, and municipality directly under the central government has formulated “management of neonatal screening” in their territories. Beijing, Shanghai, Guangdong, Shandong, Jilin, Liaoning, Henan, Hunan, Zhejiang, Heilongjiang, and other provinces in China have introduced policies related to cochlear implantation and hearing speech rehabilitation assistance, and the specific scope of assistance varies from region to region. In Shandong province, eligible pediatric patients under 18 years of age receive subsidies for imported cochlear equipment for one ear, hospitalization and surgical costs, rehabilitation, and mapping for 1 year after surgery. Postlingual deafness patients aged 8–60 years receive subsidies from the “Home of Hope” cochlear project for adults that cover the cost of a set of domestic artificial cochlea and cochlear implantation surgery for one ear.

With the development of newborn hearing screening, how to manage and use hearing screening data more conveniently and efficiently has also become a research hot topic, and the work of domestic research teams has promoted the standardization of information management of newborn hearing screening. In 2006, Zou et al*.* (47) used the phenylketonuria and congenital hypothyroid function two disease screening network to carry out newborn hearing screening simultaneously, and they found it to be a better management model for conducting newborn disease screening. In the same year, Qi et al*.* (48) suggested that using informatics to process all information obtained from the hearing screening system in a “timely, accurate, applicable and unobstructed” way is necessary. In 2007, Wu and Ma (49) proposed to set up all the information of the UNHS project as a unified web-based database, which can achieve more effective follow-up. In the same year, Liu et al. (50) developed a UNHS network database in Zhuhai, Guangdong Province, based on a UNHS data management and follow-up system which the team developed in 2009, which solved the difficulty of hearing screening follow-up (51). In 2010, the team mentioned above updated the system to achieve the functions required for integrated information management, such as resource sharing, quality control, statistical reporting, intelligent follow-up, deafness intervention, and language rehabilitation (52). In 2013, they promoted the application of the UNHS network system in primary hospitals, and the rates of the initial hearing screening, diagnosis, follow-up, and intervention improved (53). In 2015, Mai et al*.* (54) constructed a provincial and regional information management platform for newborn hearing screening. The construction of the municipal newborn hearing screening network system and the promotion of the provincial information management platform have served as an example for the establishment of a national UNHS information management platform.

### 3.3. Quality control of universal newborn hearing screening

The systematicity and completeness of the entire process of initial screening, rescreening, diagnosis, and intervention of the newborn hearing screening project play a crucial role in the quality control of the screening. In 2007, Nie et al. (55) suggested that simultaneous audiovisual screening of newborns is feasible and effective in the monitoring and prevention of hearing loss and ocular disease. In 2008, Tao et al. (56) established a quality control system for UNHS in Chengdu, Sichuan Province, and found that it could improve the coverage rate of UNHS and achieve the goal of timely intervention. In 2011, Han and Wang (57) pointed out that the UNHS program should be led by the health administration and supported by collaborating hospitals, maternal and child healthcare institutions, and rehabilitation departments to achieve successful implementation and systematic management of newborn hearing screening, diagnosis, intervention, and rehabilitation. In the same year, Wu and Huang (58) reported that diagnosis, treatment, intervention, rehabilitation, and follow-up after the hearing screening are highly disconnected and that the quality of the screening is difficult to control. He pointed out that multidisciplinary collaboration and communication should be strengthened. In 2010, the study by Yao et al. (59) showed that all provinces in the eastern region had established an information reporting system and well-developed guidelines for supervision, guidance, and quality control of the UNHS, while those in the central region were deficient. Moreover, most provinces in the western region had not yet developed quality control systems, suggesting that improving organizational management is an effective strategy to promote the development of hearing screening (59).

In 2016, Xu et al. (60) showed that the integrity and reliability of UNHS data could be quality controlled by the Zhengzhou Newborn Hearing Screening Network of Henan Province. In the same year, the research conducted by Tian et al. (61) revealed that some hospitals had substandard initial screening and rescreening rates and excess initial screening failure rates, pointing out the necessity of strengthening UNHS quality control. Later, in 2019, they reported a low screening coverage rate, suggesting the importance of strengthening UNHS education and advocacy, accurate entry of screening data into the information system, and enhanced follow-up of newborns who failed the UNHS (62). Then, in 2021, Tian et al. (63) reported a UNHS coverage rate of 95.26% in local midwifery facilities, which met the requirements of the former National Ministry of Health, but the screening facilities failed to obtain diagnostic results and management schemes for newborns who failed rescreening and were referred for hearing loss diagnosis, resulting in an incomplete data chain. A multicenter study showed that the initial screening coverage rate in 2016 and 2017 was 94.96% and 96.10%, respectively (64). In 2009, Huang et al. conducted a research on 12,638 infants who were born in nine counties and found that 85.8% of newborns had been screened (65). Thus, coverage improved from 85.8% to 96.10% in 9 years, demonstrating notable achievements in newborn hearing screening. From 2014 to 2016, the former National Health and Family Planning Commission organized larger public health service programs for children to train healthcare providers in impoverished areas on the prevention and control of birth defect, including newborn hearing screening, further improving the coverage, and promoting the standardized implementation of the newborn hearing screening program. Furthermore, to implement the outline for “the Development of Chinese Children (2011–2020)” and the spirit of “the Central Conference on Poverty Alleviation and Development” and to identify newborns with hearing loss as early as possible in impoverished areas, in 2012, the former Minister of Health of the People's Republic of China and China Disabled Persons’ Federation initiated a project to screen newborns in impoverished areas. The project utilized special subsidies from the central government to provide 70 RMB per case of newborn hearing screening in 21 provinces. The implementation of the project has played a positive role in increasing the coverage rate of newborn hearing screening in impoverished areas (66).

In summary, the above studies illustrated the importance of establishing a quality control index system for newborn hearing screening and diagnosis to strengthen UNHS quality control. In 2018, the “Guideline for the early diagnostic evaluation and intervention of hearing loss in infants” was developed, which contains diagnostic criteria, principles, methods and comprehensive assessments, intervention guidelines, and methods and effect evaluations to further standardize the diagnosis and intervention of hearing loss in early childhood (45). In 2021, Zeng et al. (67) constructed a quality control scale for newborn hearing screening and diagnosis and found that the scales have good internal consistency reliability, partial reliability, and structural validity and can be used to evaluate intelligence in newborn hearing screening and diagnosis. In the same year, Wen et al. (68) applied the Delphi method to construct a quality control index system for newborn hearing screening and diagnosis, which is expected to improve the quality control level of newborn hearing screening and diagnosis nationwide.

### 3.4. Concurrent newborn hearing and genetic screening

In 2006, Morton and Nance (69) pointed out the limitations of newborn hearing screening and first proposed the concept of concurrent newborn hearing and deafness genetic screening. In 2007, Wang et al. (70) conducted a preliminary research on the protocol and strategy for the simultaneous screening of newborn hearing and deafness genes. In 2012, Beijing launched a demonstration project of concurrent newborn hearing and deafness genetic screening. The project screened for nine mutations in four common deafness-related genes, such as c.235delC (p.Leu79Cysfs*3), c.299_300delAT (p.His100Argfs*14), c.176_191del16 (p.Gly59Alafs*18), and c.35delG (p.Gly12Valfs*2) in the *GJB2* gene (MIM: 121011); c.919-2A > G and c.2168A > G (p.His723Arg) in the *SLC26A4* gene (MIM:605646); m.1555A > G and m.1494C > T in *mtDNA12SrRNA* (MIM: 561000); and c.538C > T (p.Arg180*) in the *GJB3* gene (MIM: 603324) (71). Currently, commonly used techniques for deafness genetic screening include microarray, PCR-fluorescent probe, fluorescent PCR, fluorescent PCR melting curve, PCR and flow-through hybridization, amplification refractory mutation system, PCR melting curve, combined probe anchoring polymerization sequencing, flight mass spectrometry sequencing, screening for known deafness-causing variants based on next-generation sequencing technology, and so on.

In 2012, “330 Questions for Concurrent Newborn Hearing and Deafness Gens Screening,” which was coedited by Wang et al., was published in China. In 2015, Huang and Han (72) proposed the importance of establishing a quality control index system for newborn deafness genetic screening. Currently, many deafness genetic screening products with different coverage of genes and pathogenic variants are identified. The selection of screening variants for common deafness genes is relatively standardized; however, the selection of pathogenic variants of uncommon deafness genes varies widely among the products based on different databases. In principle, deafness screening variants should be selected with clear deafness pathogenicity and high allele frequencies based on large-scale molecular epidemiological surveys of deaf populations. At present, the National Medical Products Administration batch numbers have been obtained for the variants in deafness gene screening kits, as presented in Table 1 (73).

**Table 1:** Pathogenic variants included in deafness genetic screening.

Gene	Inheritance pattern	Nucleotide change	Protein change	Frequency in Chinese population
*GJB2* NM_004004.6	Autosomal recessive	c.235delC	p.Leu79Cysfs*3	0.0180
		c.299_300delAT	p.His100Argfs*14	0.0050
		c.176_191delGCTGCAAGAACGTGTG	p.Gly59Alafs*18	0.0012
		c.35delG	p.Gly12Valfs*2	0.0001
		c.167delT	p.Leu56fs	0
		c.109G > A	p.Val37Ile	0.0693–0.1250
		c.257C > G	p.Thr86Arg	0.0003
		c.508_511dupAACG	p.Trp172Thrfs*40	0.0003
		c.427C > T	p.Arg143Trp	0.0003
		c.35dupG	p.Val13Cysfs*35	0.0006
*SLC26A4* NM_000441.2	Autosomal recessive	c.919-2A > G	Aberrant splicing	0.0134
		c.2168A > G	p.His723Arg	0.0027
		c.1174A > T	p.Asn392Tyr	0.0008
		c.1226G > A	p.Arg409His	0.0010
		c.1229C > T	p.Thr410Met	0.0008
		c.1975G > C	p.Val659Leu	0.0013
		c.2027T > A	p.Leu676Gln	0.0007
		c.589G > A	p.Gly197Arg	0.0003
		c.1707 + 5G > A	Aberrant splicing	0.0003
		c.281C > T	p.Thr94Ile	0.0003
		c.2162C > T	p.Thr721Met	0.0001
		c.916dupG	p.Val306Glyfs*24	
12SrRNA	Mitochondrial	m.1494C > T		0.0002
		m.1555A > G		0.0021

In 2019, Wang et al. (74) conducted a research on a nationwide population cohort of 1,172,234 newborns, which showed that genetic screening detected 13% more hearing-impaired infants than hearing screening alone and identified 0.23% of the newborns who were predisposed to preventable ototoxicity undetectable by hearing screening. In the same year, Dai et al*.* (75) conducted a concurrent hearing and genetic screening follow-up study of 180,469 neonates in Beijing and found that 25% of infants with pathogenic combinations of *GJB2* or *SLC26A4* variants and 99% of infants with a m.1555A > G or m.1494C > T variant passed the routine newborn hearing screening test. In the same year, Ruan et al*.* (76) reported that 20% of newborns with biallelic mutations passed the newborn hearing screening test. The research indicated that genetic deafness screening was an important supplement to newborn hearing screening. In 2020, Wen et al. (77) analyzed the status of newborn genetic screening in 2016 and 2017 in multiple regions of China, suggesting that genetic screening is more widely carried out in the eastern region of China, while it has yet to be promoted in the central and western regions. In 2022, a large cohort study of 3,555,336 newborns reported by Zhang et al. (78) showed that *GJB2* gene c.235delC was the most common variant in the Chinese newborn population, followed by *SLC26A4* gene c.919-2A > G, differing from those in the Ashkenazi Jewish, European, and American populations. In 2023, the current authors’ research team ascertained the advantage of a screening chip including 15 variants of four genes and found that 71.43% of newborns with two variants of the *SLC26A4* gene were screened for newly added mutations and 28.57% of newborns with two variants of the *SLC26A4* gene passed the hearing screening, suggesting that a screening chip including 15 variants of four genes was superior at early detection of hearing loss, especially in early identification of newborns with deafness-causing mutations in the *SLC26A4* gene (71). The genetic screening identified additional children who will possibly benefit from early intervention and informed at-risk newborns and their maternal relatives of increased susceptibility to ototoxicity (74). Tertiary prevention of deafness birth defects could be achieved through concurrent newborn hearing and genetic screening, preventing disability due to deafness. Prenatal or pre-embryonic genetic testing can radically stop the transmission of deafness in the family line and prevent deafness in newborns at birth. Preconception blockade in at-risk populations, pregnancy blockade in at-risk populations, and postnatal blockade in at-risk newborns could also reduce the incidence of hereditary deafness (79).

With the implementation of newborn deafness genetic screening, a series of clinical guidelines have been issued. In 2020, the Medical Genetics Branch of the Chinese Medical Association issued the “Clinical Guidelines for Hereditary Non-syndromic Deafness,” including the prevalence of non-syndromic deafness in the population, the pathogenic gene profile, and information on the correlations between clinical phenotypes and genotypes, providing guidance for clinical consultants and otologists (80). In 2021, the Chinese Multi-center Clinical Research Collaboration Group on Genetic Screening and Diagnosis of Deafness developed the Genetic Deafness Screening Specification to standardize the genetic deafness screening and postscreening workflows in China to more effectively achieve early diagnosis, treatment, and prevention of deafness in newborns (73). All children who “Refer” in genetic screening and those who “Pass” in genetic screening but “Refer” in hearing screening should be referred to a qualified physician for further diagnosis and genetic counseling. Audiology, medical imageology, and genetic diagnosis of deafness should be performed at a medical institution qualified in additional diagnostic procedures. In addition, more accurate genetic counseling and rehabilitation guidance should be provided (73). In 2023, the collaborative group mentioned above issued the “Clinical Practice Guidelines for the Genetic Diagnosis and Counseling of Hearing Loss in China (2023)” to clarify the value of genetic diagnosis in the treatment of deafness and standardize the process of genetic deafness diagnosis in China (79).

To date, concurrent newborn hearing and deafness genetic screening in China has entered a more mature stage of development and has clinical importance for the management of congenital deafness and prevention of ototoxicity.

### 3.5. Expanding from newborn hearing screening to pediatric hearing care

Newborn hearing screening allows for the early detection of congenital hearing loss. Some children who pass the UNHS test present with delayed or acquired hearing loss as they grow, and pediatric hearing screening can detect such children early (81). Huang et al. (82) also reported that pediatric hearing screening can detect and diagnose hearing loss in preschool children earlier and is an important complement to UNHS. In 2013, Lü et al. (83) found that a certain percentage of preschool children aged 3–6 years who had passed the UNHS test showed delayed hearing loss without obvious symptoms, and the implementation of hearing screening in preschool children could detect those with delayed hearing loss at an early stage. Later, in 2014, they developed an intelligent system that could be applied to children's hearing screening (84). It has been reported that late-onset hearing loss in children deserves particular attention (85). Tao and Yang also indicated that systematic and standardized hearing screening in the pediatric population is another important task after UNHS (86). Xiao et al. analyzed the association between hearing thresholds and emotional problems in school-aged children and found that emotional symptoms were positively associated with average hearing thresholds (87). Therefore, early detection of hearing loss in children is of great importance.

In 2011, guidelines endorsed by the American Academy of Audiology recommended screening school-aged children in preschool, kindergarten, and grades 1, 3, 5, and either 7 or 9 (88). Since the 1990s, research on pediatric hearing screening has been carried out in Beijing, Shanghai, Wuhan, and other cities in China. In 2003, the Beijing Municipal Health Bureau issued the “Beijing Municipal Hearing Screening and Diagnosis Management Measures for Children Aged 0–6 Years” and took the lead in carrying out hearing screening and providing hearing healthcare for children aged 0–6 years, laying the foundation for nationwide ear and hearing care work for children aged 0–6 years. In 2005, the Beijing Children's Hearing Care Expert Steering Group summarized the experience of early hearing detection and intervention for children aged 0–6 years in Beijing, further standardized hearing care work for children, and conducted preliminary exploration and training for the development of childhood hearing care nationwide (89). After years of clinical practice, in 2013, the “Technical Specification for Children's Ear and Hearing Care” was promulgated. This specification stated that hearing screening should be carried out in children aged 0–6 years and specified the methods of implementation, referral processes, work requirements, and assessment indicators, providing standardization of pediatric hearing screening nationwide (12).

In conclusion, hearing screening and hearing care for children aged 0–6 years is an important supplement to UNHS. It is an important method for the early detection of delayed hearing loss in children, but due to the complexity of hearing screening in children, the sensitivity and specificity of screening methods for different age groups have yet to be explored. Therefore, while increasing the popularization of hearing screening for children in the future, it is necessary to develop screening methods and procedures for different age groups to improve the quality of screening and promote the feasibility of carrying out screening at the grassroots level.

## 4. The future stage of newborn hearing screening

Newborn hearing screening in China has been widely carried out nationwide, and how to further develop hearing screening in the future is worth considering. The concept of hearing screening and hearing care across the whole life course should be implemented to further enrich the content of screening. In addition, universal cytomegalovirus (CMV) infection screening and standardized hearing screening for children at the county level should be conducted soon to continuously improve the hearing screening system and the quality of screening.

### 4.1. Hearing screening and hearing care across the whole life course

In 2021, the World Health Organization released the World Hearing Report, which noted that each individual has a unique hearing trajectory that is shaped by diverse influences experienced throughout the life course (1). Therefore, the World Hearing Report first established hearing loss across the life course as a public health priority among policymakers. For Chinese scholars to understand the call of the World Health Organization with regard to hearing healthcare across the life course, Han Demin organized experts to translate the World Hearing Report into Chinese, which was published by the People's Medical Publishing House (90). Therefore, how to establish and improve hearing screening across the life course and the hearing care system to achieve early detection, diagnosis, and intervention of hearing loss in the entire population has become the main goal of our future work.

With regard to newborn hearing screening, the main methods used for the genetic diagnosis of hereditary deafness include gene chip, Sanger sequencing, capture sequencing of target deafness genes (i.e., second-generation sequencing of known deafness gene panels), whole-exome sequencing (WES), and whole-genome sequencing (WGS) (79). Furthermore, China has issued a series of policies regarding children's health in recent years. In 2018 and 2021, the national birth defect prevention and control personnel training program training syllabus (for trial implementation) and the national birth defect prevention and treatment personnel training program training syllabus (2021 version) were issued. Later, in 2021, the National Occupational Skills Standards for Birth Defects Prevention and Control Counselors (2021 Edition) were issued. The above policies include all aspects of hearing birth defect prevention and control, providing a guarantee for the training and reserve of vocational skill personnel for hearing birth defect prevention and control nationwide and comprehensive talent support for hearing healthcare across the whole life course. In addition, the National Health Commission of the People's Republic of China issued the Healthy Children Action Enhancement Plan (2021–2025) in 2021 to further improve the level of eugenic services and promote children's health.

In terms of early detection of delayed hearing loss, attention should be given to the expansion of genetic screening deafness variants. Most domestic studies have shown that the *GJB2* gene p.V37I mutation has a high frequency in the Chinese population. Chen et al. (91) reported that the rate of the biallelic p.V37I mutation was 0.528%. p.V37I mutations were detected in 2.8%–8.2% of the normal Chinese Han population and 6.7%–15.05% of the Chinese deaf population (92–96). Recently, Chen et al. (91) reported that the p.V37I variant in *GJB2* is associated with steadily progressive hearing loss with an increasing incidence over the life course. Most individuals with the biallelic p.V37I variant may develop significant hearing loss in adulthood and can benefit from early diagnosis and intervention through widespread genetic screening. In 2023, the *GJB2* gene p.V37I mutation will be incorporated into deafness genetic screening in Beijing. This measure will help in early detection of delayed hearing loss.

With regard to adult hearing health, hearing screening can be undertaken using technology-based solutions. Screening is facilitated by the development of mobile-based software applications that provide cost-effective and easy-to-use tools. The hearWHO app is based on validated digits-in-noise technology and gives the general public access to a free, validated hearing screener to check their hearing status and to monitor it over time. The easy-to-use app clearly displays the users’ results and maintains a personalized record of their hearing status over time. It is available in both Android and iOS formats (1). An expert consensus on hearing screening in the Chinese health checkup population was released in 2016, which clarified the different hearing screening standards for people under 35 years old and those over 36 years old, and established a unified screening technique and standardized pathway for the healthy examination population in China (97). With regard to hearing health in older age, an expert consensus on the diagnosis and intervention of hearing loss in elderly individuals was released in 2019. This consensus clarified the screening methods, screening process, and intervention and rehabilitation training modalities for hearing loss in older age, providing guidance for early detection and early intervention in individuals with age-related hearing loss (98).

In the future, we need to learn from previous experiences in newborn hearing screening and pediatric hearing healthcare. We should conduct more in-depth research to further improve the hearing healthcare workflow for preschool and school-aged children. We could also expand early hearing screening for adults and elderly individuals, as well as for all people at higher risk of hearing loss due to exposure to noise, ototoxic chemicals, and ototoxic drugs. Otologists, audiologists, and other related interdisciplinary workers should explore the technical procedures and programs for hearing screening and hearing care throughout the life course that are appropriate for the national conditions in China to help in the development of hearing healthcare in the country.

### 4.2. Concurrent newborn hearing, genetic, and congenital cytomegalovirus infection screening

Delayed hearing loss in children attributed to congenital cytomegalovirus (cCMV) infection has received attention in recent years. The World Hearing Report stated that certain infections, such as CMV infection, occurring in the newborn period may be attributed to pathogens that have a direct effect on the auditory system (1). Cytomegalovirus infection is considered the common cause of SNHL, with a prevalence rate of 0.7% in newborns in China (99), and approximately 10%–25% of SNHL cases in children are caused by cCMV infection (100). Studies have shown that 10% of children with cCMV present with obvious clinical symptoms at birth and 90% are born without specific clinical signs or symptoms. Approximately 30%–60% of children with symptomatic infection and 10%–15% of children with asymptomatic infection develop SNHL, so the early detection of such infections in children is particularly important (101). The diagnostic rate of bilateral profound SNHL using a combined genetic deafness test and CMV DNA detection test was reported to be 46.4% (102). In 2018, Moteki et al. (103) reported that dried blood spot (DBS) screening for cCMV may be sufficient in a clinical setting and offers a realistic and feasible option for universal mass screening. Lu et al. (104) conducted concurrent hearing, genetic, and CMV screening in 1,716 newborns in Taiwan and found that 100% of the newborns who tested positive for CMV passed the UNHS test at birth. The study confirmed the feasibility of concurrently performing hearing, genetic, and CMV screenings in newborns and provided evidence that the incorporation of these screening tests could potentially identify an additional subgroup of infants with impaired hearing who would not possibly be detected by UNHS programs (104).

A universally combined newborn hearing, genetic deafness, and cCMV screening has not yet been implemented in Mainland China, and cCMV screening is rarely performed, which perpetuates the lack of awareness of cCMV infection and related conditions. However, large population-based cohort studies from China have shown that more than 98% of cCMV-positive newborns are born to women who are seropositive in early gestation (105). Fang et al. (106) reported that maternal human CMV infection during pregnancy accounted for 2.92% of the etiology of newborn hearing impairment in Guangdong Province, China. In 2019, the “Guidelines for Screening and Clinical Intervention of Congenital Cytomegalovirus Infection” provided detailed recommendations on common problems in the screening, diagnosis, intervention, and treatment of human cytomegalovirus (HCMV) infection. The guidelines also advocate that neonatal cytomegalovirus screening should be universal to enable early detection of cCMV-infected infants for the early diagnosis and intervention of organ system damage, such as SNHL and developmental delays (107). Over the previous four decades, China has issued several guidelines, technical specifications, and expert consensuses related to newborn hearing screening, which have greatly promoted the development of newborn hearing screening in China. Table 2 briefly summarizes the contents of the guidelines.

**Table 2:** Guidelines, technical specifications, and expert consensuses on newborn hearing screening in China.

Stage	Year	Title	Main recommendations
Past	2004	Technical Specification for Newborn Hearing Screening (9)	1. A two-stage screening process is implemented by OAEs or AABRs: those who fail the initial screening before discharge from the hospital are rescreened within 42 days and referred to a hearing testing center. 2. Newborns with high-risk factors in conjunction with auditory behavior observations should be followed up every 6 months for 3 years even if they pass the screening test.
Present	2010	Technical Specification for Newborn Hearing Screening (2010 Edition, No. 96) (11)	1. Normal newborns are screened in two stages: 48 h after birth and before discharge. Those who fail and those who miss screening should be rescreened within 42 days. Those who do not pass the rescreening test should be referred for hearing diagnosis within 3 months. 2. For NICU newborns, the infant should be screened by AABRs before discharge. Those who fail will be referred directly for hearing diagnosis. 3. Newborns with risk factors for hearing loss, even if they pass the newborn hearing screening test, should be followed up at least once a year for 3 years.
	2013	Technical Specification for Children's Ear and Hearing Care (12)	1. After hearing screening in the neonatal period, children aged 0–6 years are managed in the healthcare system. 2. Ear and hearing care is provided in conjunction with health screening. 3. The priority ages for hearing screening are 6, 12, 24, and 36 months of age.
	2018	Guideline for the Early Diagnostic Evaluation and Intervention of Hearing Loss in Infants (45)	Normal range of hearing: 1. Normal tympanogram on the conductance test (including 1,000 Hz sound detection). 2. V-wave response threshold of ≤35 dB nHL on the click sound ABR test. 3. The amplitude of each frequency of DPOAEs is within the normal range, and the signal-to-noise ratio is ≥6 dB. The correlation coefficients of TEOAEs are greater than 50% for each frequency band and greater than 70% for the total correlation coefficient. 4. Hearing threshold of behavioral hearing assessment is in the normal range.
	2020	Clinical Practice Guidelines for Hereditary Non-syndromic Deafness (80)	1. Up to September 2018, 110 genes related to non-syndromic deafness have been identified, including 45 genes related to DFNA, 70 genes related to DFNB, 10 genes related to both DFNA and DFNB, and 5 genes related to DFNX. 2. China Deafness Genetic Research Consortium (http://cdgc.eargene.org) is recommended as a starting query for Chinese or East Asian deafness cases.
	2021	Genetic Deafness Screening Specification (73)	1. Newborns: All newborns aged 3 days after the completion of heel blood or cord blood collection. 2. Individuals with normal hearing, including those with a requirement for marriage and childbirth. • Deafness genetic screening is recommended before marriage, pregnancy, or early pregnancy. It is recommended that couples undergo screening at the same time and subsequent genetic diagnosis as necessary to improve the accuracy and efficiency of screening or diagnosis. 3. Deaf patients: It is recommended that deaf patients undergo direct genetic diagnosis of deafness rather than genetic screening.
	2022	Chinese Clinical Practice Guideline of Auditory Neuropathy (version 2022) (43)	1. Newborns in the NICU should first undergo AABR screening. 2. Diagnostic criteria for infantile auditory neuropathy: • Children <3 years of age who often pass the newborn hearing screening (OAEs), rescreening, and diagnostic OAE tests are normal, and CM can be elicited normally, but the ABR often shows a waveform with no significant differentiation or severe abnormalities. Genetic diagnosis may reveal pathogenic genetic variants, but the imaging examination does not suggest posterior cochlear lesions or abnormal auditory nerve development.
	2023	Clinical Practice Guideline for the Genetic Diagnosis and Counseling of Hearing Loss in China (2023) (79)	1. Deafness genetic diagnosis is indicated for patients with congenital deafness, delayed deafness, syndromic deafness, normal hearing with a family history of deafness, and unsuccessful deafness genetic screening results. 2. Methods of genetic deafness diagnosis include gene chip, Sanger sequencing, targeted deafness gene next-generation sequencing, whole-exome sequencing, and whole gene sequencing. 3. For hereditary deafness with a clear phenotype and genotype, specific genes can be tested. For hereditary deafness with an unclear phenotype and genotype, “Definitive” or “Strong” genes can be tested and evaluated by ClinGen as a priority. 4. For detected variants, pathogenicity should be interpreted according to the ACMG classification criteria.
Future	2019	Guidelines for Screening and Clinical Intervention for Congenital Cytomegalovirus Infection (107)	1. There should be universal newborn HCMV screening for the early detection of congenital HCMV infection in infants. 2. Congenital HCMV infection is diagnosed by detecting the HCMV-DNA content of saliva or urine samples from newborns within 2 weeks after birth.
	2021	National Occupational Skills Standards for Birth Defects Prevention and Control Counselors (2021 Edition)	Target population: Counselors are engaged in birth defect prevention and control publicity, education, counseling, guidance, and providing birth defect occurrence risk evidence-based information, genetic counseling, solution recommendations, prevention and control management services, and rehabilitation counseling.

ABR, auditory brainstem response; DFNA, autosomal dominant deafness; DFNB, autosomal recessive deafness; DFNX, X-linked deafness; CM, cochlear microphonic; ACMG, American College of Medical Genetics and Genomics; nHL: normal hearing level.

## 5. Summary and outlook

Newborn hearing screening in China has gradually evolved from initial localized screening to nationwide universal screening and concurrent hearing and deafness genetic screening and expanded to nationwide childhood hearing screening and care. In the future, we should implement the concept of hearing screening and healthcare across the whole life course and conduct universal congenital cytomegalovirus infection screening to achieve early detection of congenital deafness associated with congenital cytomegalovirus infection. Eventually, a comprehensive, full-coverage, whole life course hearing screening and intervention system should be developed.
